# Sleeping Sickness: A Tale of Two Clocks

**DOI:** 10.3389/fcimb.2020.525097

**Published:** 2020-10-02

**Authors:** Filipa Rijo-Ferreira, Joseph S. Takahashi

**Affiliations:** ^1^Department of Neuroscience, Peter O'Donnell Jr. Brain Institute, University of Texas Southwestern Medical Center, Dallas, TX, United States; ^2^Howard Hughes Medical Institute, University of Texas Southwestern Medical Center, Dallas, TX, United States

**Keywords:** circadial rhythm disorders, circadian, parasite, infectious disease, sleep

## Abstract

Sleeping sickness is caused by a eukaryotic unicellular parasite known to infect wild animals, cattle, and humans. It causes a fatal disease that disrupts many rhythmic physiological processes, including daily rhythms of hormonal secretion, temperature regulation, and sleep, all of which are under circadian (24-h) control. In this review, we summarize research on sleeping sickness parasite biology and the impact it has on host health. We also consider the possible evolutionary advantages of sleep and circadian deregulation for the parasite.

## *Trypanosoma brucei* Causes Sleeping Sickness: Epidemiology and Clinical Features

**Figure 1:**
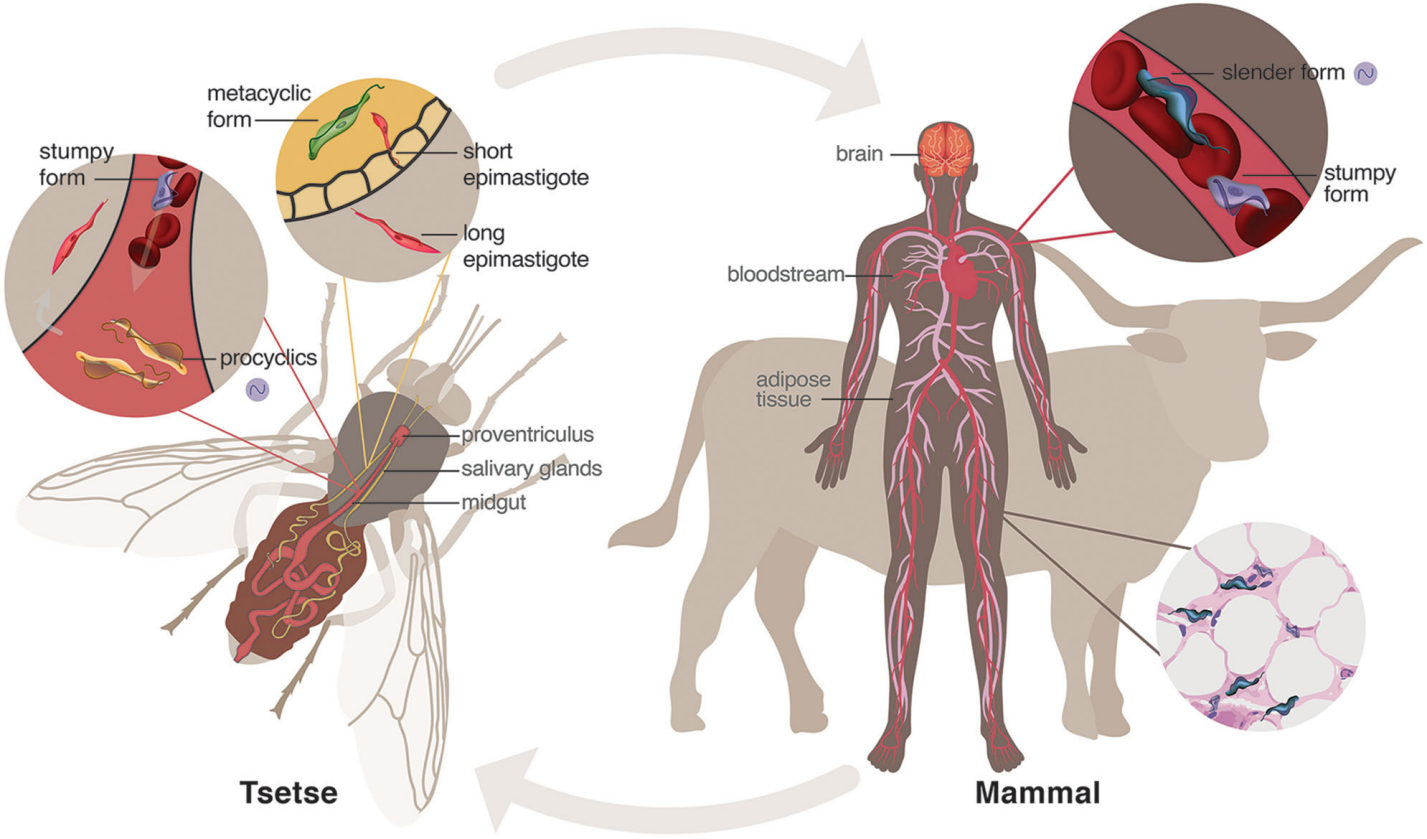
Life cycle of *Trypanosoma brucei*. When a tsetse fly vector has a blood meal from an infected human, parasites enter the midgut where mammalian bloodstream stumpy forms sense the lower temperature and different nutrient environment and differentiate into procyclic forms. These will further differentiate into epimastigotes that journey to and populate the salivary glands. The short epimastigotes in the salivary glands goes through a final differentiation in the fly into the infective metacyclic forms, which are injected during the next blood meal of the fly into the mammalian host. In the bloodstream of the mammalian host, metacyclic parasites differentiate into bloodstream forms, consisting of dividing slender forms and cell-cycle arrested (non-dividing) stumpy forms. These parasites can invade and hide within niche conditions in tissues, such as brain and adipose tissue. Purple cycling circle indicates the parasite forms in which endogenous circadian rhythms have been identified (Rijo-Ferreira et al., 2017a).

**Figure 2:**
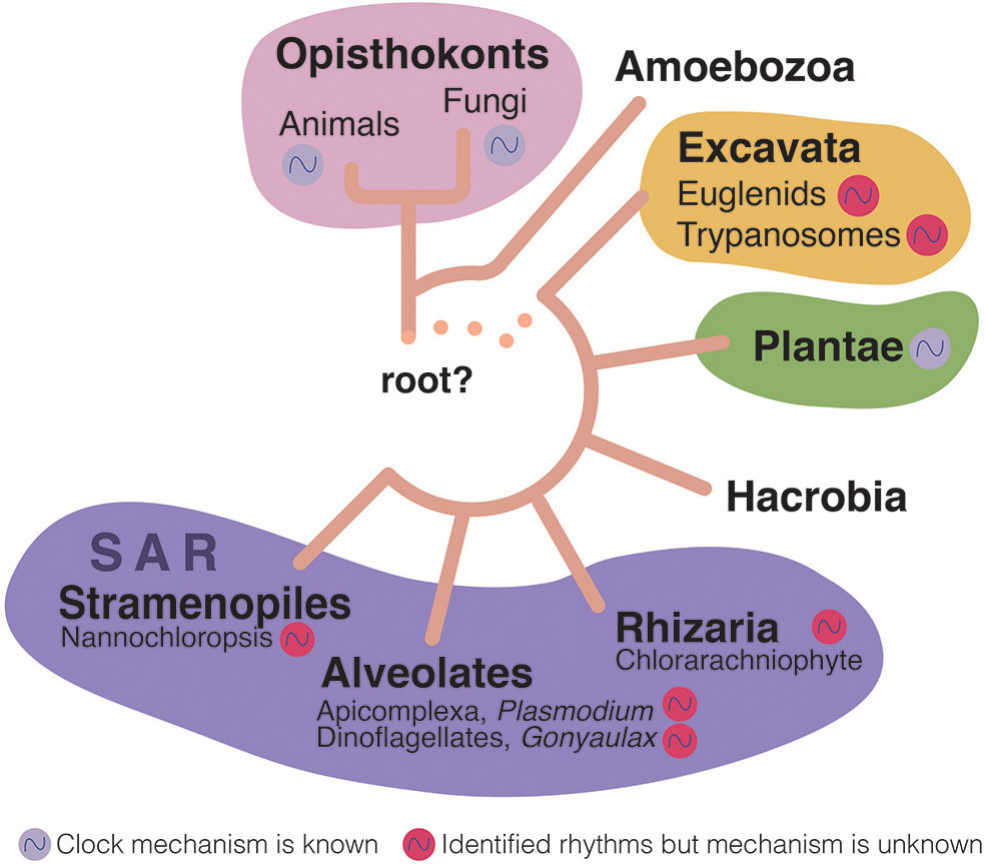
Eukaryotic tree of life (Adl et al., 2019; Burki et al., 2020). Rhythms have been identified in most of the subkingdoms of eukaryotes (colored cycling circles near the organism group). However, only a few of these rhythms have been shown to be endogenous to the organisms themselves (although possibly all these organisms have endogenous clocks), and in even fewer the actual circadian clock genes have been identified (purple cycling circle). Euglenids are free-living, aquatic flagellates; Trypanosomatids are eukaryotic parasites; Chlorarachniophytes are a small group of marine algae; Apicomplexa is a group that includes many parasites including *Plasmodium*, which causes malaria [it has been recently shown to have an endogenous clock (Rijo-Ferreira et al., 2020; Smith et al., 2020)]; *Gonyaulax* is a genus of dinoflagellates that are aquatic organisms with two separate flagella; Nannochloropsis are microalgae living in freshwater and seawater that are related to diatoms and brown algae.

Human African trypanosomiasis (HAT), best known as sleeping sickness, is a fatal infectious disease caused by the unicellular parasite, *Trypanosoma brucei*. It is transmitted via the bite of a obligate blood-feeding tsetse fly (*Glossina* spp.) and is endemic to sub-Saharan Africa, where the tsetse fly thrives (WHO, 2013) (Figure 1). Trypanosomes are evolutionarily distant from animals, fungi, and plants (Cavalier-Smith, 2010) (Figure 2), as they belong to a very particular class (Kinetoplastida) within the phylum Euglenozoa, that is characterized by its unique mitochondrial DNA: the kinetoplast DNA (kDNA). Trypanosomatids are kinetoplastids that have acquired an obligatory parasitic lifestyle (Lukes et al., 2014), and both *Trypanosoma* and *Leishmania* genera contain species that spend their lives between a mammalian host and insect vector. *T. cruzi*, which causes Chaga's disease, and *Leishmania* spp., which cause Leishmaniasis, both develop intracellularly at some stage of their life cycle, whereas *T. brucei* lives exclusively as an extracellular parasite (Lukes et al., 2014).

Two *T. brucei* subspecies are pathogenic to humans: *T. b. gambiense* and *T. b. rhodesiense*. *T. b. gambiense*, present in western Africa, causes ~97% of current cases, whereas *T. b. rhodesiense*, in eastern Africa, is less prevalent, causing only 3% of cases (Franco et al., 2017). A third subspecies–*T. b. brucei*–is non-infective to humans and instead parasitizes domestic and wild animals, causing a disease called Nagana, which severely hampers cattle production in one-third of the African continent (WHO, 2013). The minor difference between these subspecies is that, unlike human infective trypanosomes, *T. b. brucei* cannot escape an innate immune response in humans known as trypanosome lytic factor (TLF) serum complexes (Vanhamme et al., 2003; Uzureau et al., 2013; Pays et al., 2014).

Clinically, sleeping sickness is divided into two stages. In the early stage, parasites can be found in the bloodstream and interstitial spaces of several organs (Trindade et al., 2016; Carvalho et al., 2018), after which they actively invade the central nervous system, marking the start of the late stage. *T. b. gambiense* infection is chronic, with an estimated average duration of around 3 years evenly divided between the two stages (Checchi et al., 2008). *T. b. rhodesiense* disease is usually acute, and death occurs within weeks to months (Odiit et al., 1997), possibly due to this parasite being less adapted to humans (WHO, 2013). Because of the widespread nature of the infection, patients experience myriad symptoms, including chronic and intermittent fever, headache, pruritus, lymphadenopathy, and (infrequently) hepatosplenomegaly in the early stage. In the late stage, sleep disturbances and neuropsychiatric disorders dominate the clinical presentation, giving rise to the disease's common name of “sleeping sickness” (Brun et al., 2010). It is important to note that this infection does not cause hypersomnia, as the total amount of time spent asleep by these patients is similar to healthy individuals. Instead, sleeping sickness alters sleep architecture and the timing at which sleep occurs (Buguet et al., 2005) (see below for more details).

Since the late 1990's, in an international coordinated effort led by the World Health Organization, control and surveillance programs have been reinforced, which has resulted in a 30-fold drop in sleeping sickness cases to <10,000 new cases per year (WHO, 2019). Failure of careful vector control, as occurred in the 1960's due to political instability, war, and famine, led to a rise in cases to almost half a million by the 1990's. Thus, continuous programs are required to limit spread (Kennedy, 2004). Nevertheless, as a whole, African trypanosomiasis still represents a serious health and socio-economic burden, and it is believed that a substantial number of human and animal infections remain unreported since the disease is endemic in rural areas (Beschin et al., 2014). Besides the severe life threat posed by sleeping sickness to humans, Nagana, causes around 3 million cattle deaths and total annual losses of US $4.75 billion. Nagana is a particularly heavy burden in rural areas where livestock production is the main livelihood, perpetrating poverty and underdevelopment (FAO, 2014). In addition, Nagana infected animals may even serve as a reservoir of parasites for human infection, which increases the need of further intervention (Informal Expert Group on Gambiense HAT Reservoir et al., 2018).

Sleeping sickness is lethal if left untreated; however, some sporadic cases of natural progression to asymptomatic carriage or even apparent spontaneous resolution of the infection have been reported for *T. b. gambiense* infection, resembling the *trypanotolerance* phenomena described for some African cattle species (Jamonneau et al., 2012). Unfortunately, a major obstacle for eradication of sleeping sickness is the insufficiency of existing treatments. No prophylactic treatment or vaccine are available, and so far the common drugs used to treat early and late stage sleeping sickness are not orally accessible, often very toxic, and sometimes ineffective (Jacobs et al., 2011; Buscher et al., 2017; Field et al., 2017).

## A Life of Adaptations: From Vector to Host and Back

*Trypanosoma brucei* requires two obligatory hosts to complete its life cycle: the blood-feeding tsetse fly vector and a mammalian host (Figure 1). Mammalian infection starts with a bite from an infected tsetse that inoculates cell-cycle arrested (metacyclic stage) parasites into the mammalian bloodstream and lymphatic system. These infective cells sense their new host environment and differentiate into bloodstream-form parasites that can actively replicate (slender forms) and infiltrate the interstitial spaces of several organs, including adipose tissue, skin, testes, brain, and heart (Caljon et al., 2016; Capewell et al., 2016; Trindade et al., 2016; Carvalho et al., 2018; Silva Pereira et al., 2019). There is a second bloodstream form (stumpy form) that arises from the differentiation of the slender forms and becomes cell-cycle arrested. This differentiation from slender into stumpy forms is important to constrain the number of parasites to avoid killing the host, and also because stumpy forms are transmissible to the tsetse fly, allowing the progression of the life cycle (Rojas and Matthews, 2019). Curiously, this transformation depends on the slender forms sensing each other to estimate their numbers, known as *quorum sensing*. Recently, the oligopeptide stumpy-inducing factor and the potential sensing mechanism by a G protein-coupled receptor have been elucidated (Rojas et al., 2019). Once taken up by a tsetse during a blood meal, slender forms presumably die, whereas stumpy forms differentiate into procyclic forms in the midgut (Rico et al., 2013). These, in turn, will differentiate into non-replicative mesocyclics that migrate as swarms toward the anterior part of the fly where they differentiate into long epimastigotes (Kruger et al., 2018). Epimastigotes divide asymmetrically into long and short forms, with the highly mobile long forms migrating toward the salivary glands of the tsetse, while the short epimastigotes differentiate into metacyclic forms, completing the life cycle (Buscher et al., 2017). Recent studies have characterized the beautiful dynamics of trypanosomes in the tsetse fly in detail (Schuster et al., 2017). Throughout different tissues and hosts, *T. brucei* parasites functionally adapt to their environments by adjusting their metabolism and their biology through the life cycle (Trindade et al., 2016; Smith et al., 2017; Pineda et al., 2018; Figure 1).

It is still unclear how *T. brucei* parasites invade the brain, i.e., whether they penetrate the blood-brain barrier (BBB) or whether they cross the blood-cerebral spinal fluid (CSF) barrier, or both (Bentivoglio and Kristensson, 2014; Mogk et al., 2016). One thing is clear: parasites can be observed in high numbers in the choroid plexus in animal models (Trindade et al., 2016), where blood is filtered into CSF, making this a widely-accepted entry point to the brain. Curiously, although *T. brucei* can be observed in the CSF of patients [one of the methods to diagnose late stage of infection (WHO, 2019)], trypanosomes do not survive for long in CSF (Wolburg et al., 2012). Parasites could also be crossing the BBB similarly to lymphocytes (Mulenga et al., 2001; Laperchia et al., 2016). When parasites reach the brain parenchyma they seem to concentrate in the median eminence and hypothalamic areas (Lundkvist et al., 2004; Rijo-Ferreira et al., 2018), but the brain-biogeographical and/or physiological cues parasites use remain elusive. The crossing of the BBB appears to be associated with IFN-γ signaling, as mice lacking IFN-γ or its receptor have fewer parasites in the brain parenchyma (Masocha et al., 2004). The fact that parasites can reach the brain parenchyma without BBB disruption is also supported by the fact that anti-trypanosome treatments that *cannot* cross the BBB do not cure the late stage of this disease, once parasites have reached the brain (Field et al., 2017).

## Sleep and Circadian Disruption by Sleeping Sickness Infection

**Figure 3:**
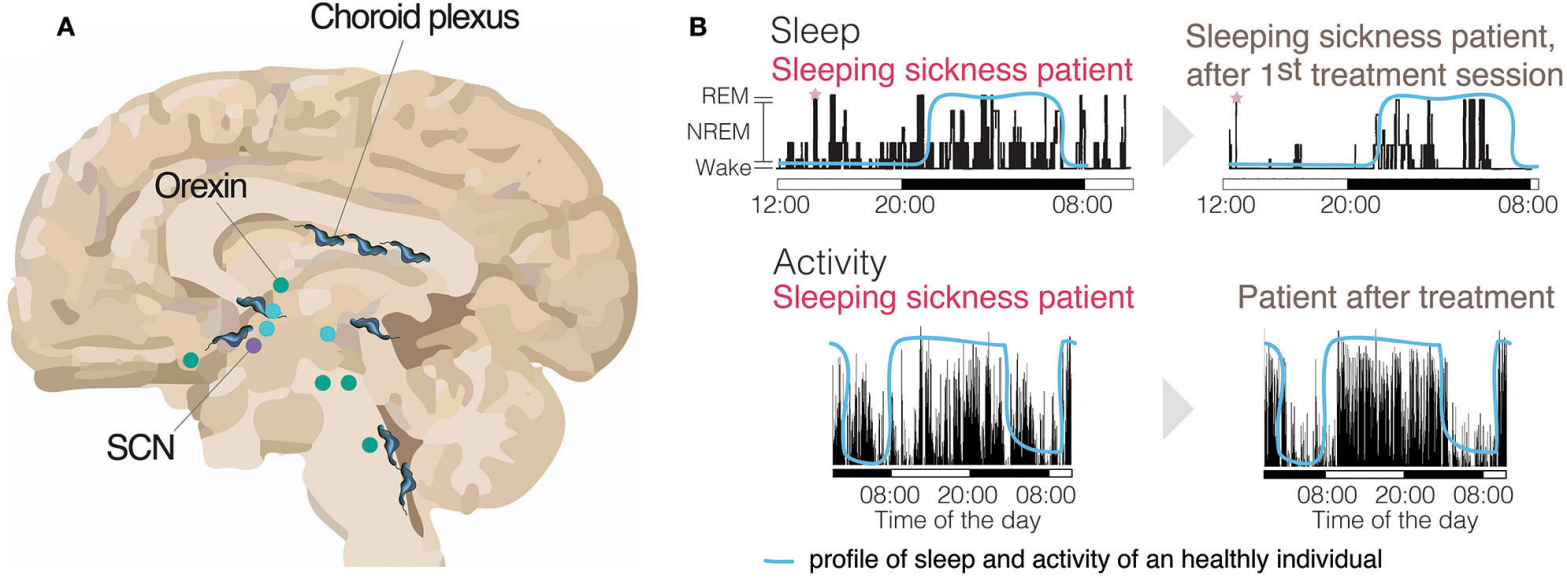
Activity and sleep disruption in sleeping sickness patients. **(A)** Brain (sagittal section) with many of the sleep-wake regulating regions identified and the circadian master clock, the Suprachiasmatic Nucleus (SCN). Represented with trypanosomes is the parasite distribution across the brain. Wake promoting brain areas are represented in green. Sleep promoting brain areas are represented in blue (Scammell et al., 2017). (**B**, top panels) Electroencephalogram (EEG) recording of a sleeping sickness patient before and after the first treatment session, showing a clear reversal of the sleeping time to the nighttime upon treatment, similar to healthy individuals (blue line) (adapted with authorization from Buguet et al., 1999). The sleeping sickness patient record shows both increased sleep during the daytime and transitions from wake to REM state (SOREM), highlighted with pink stars. (**B**, bottom panels) An activity record of a sleeping sickness patient, measured by actigraphy, using activity watches. The same patient had his/her activity measured both before and after pentamidine treatment and show clear reversal of the abnormal activity profile after treatment (adapted with authorization from Njamnshi et al., 2012). The blue line represents the normal profile of a healthy individual.

When we sleep and how much sleep we need depend on the time of day and how long we have been awake. Sleep is controlled by crosstalk between the circadian clock and other brain regions involved in regulating the homeostatic sleep process. The circadian clock is a self-sustained ~24 h molecularly driven oscillation that regulates multiple physiological aspects of mammalian biology, including sleep (Rijo-Ferreira and Takahashi, 2019). Although a circadian clock exists in each cell in the body, the suprachiasmatic nucleus (SCN) of the hypothalamus is the central brain region required for keeping clocks in the body in sync. The sleep homeostat tracks the need for sleep, which increases during wake and decreases during sleep (Scammell et al., 2017). Sleep, itself can be divided into an initial deep sleep (NREM, non-rapid eye movement) followed by a lighter sleep when dreaming occurs (REM, when brain activity is similar to being awake but without muscle tone). Unlike the circadian system, for which much of the central timekeeping mechanism is understood (Takahashi, 2017; Rijo-Ferreira and Takahashi, 2019), we still lag behind in our understanding of how the sleep homeostat is regulated, potentially because it is a more widespread, complex, and interconnected system. Incredibly, multiple areas of the brain (basal forebrain, hypothalamus, thalamus, locus coeruleus, brainstem, etc.) and numerous neuronal systems (serotonin, adenosine, orexin, GABA, histamine, etc.) are involved in sleep regulation (Imeri and Opp, 2009; Scammell et al., 2017; Figure 3).

Inflammation can affect both the homeostatic and circadian systems. Pro-inflammatory cytokine responses to stimuli can lead to a reduction in amplitude of circadian clock gene expression that also correlates with a decrease in movement, leading to what is known as “sickness-like behavior” (Cavadini et al., 2007; Leone et al., 2012; Rijo-Ferreira et al., 2018). Inflammation has also been shown to interfere with sleep. TNF-α and IL-1β pro-inflammatory cytokines regulate fever in the response to an infection (even common infections like influenza), and are both somnogenic, meaning they increase NREM sleep (Krueger et al., 2001; Imeri and Opp, 2009). Other humoral factors, such as the pro-inflammatory cytokines IFN-γ, IL-2, IL-6, IL-15, and IL-18, as well as prostaglandin D2 (PGD2), which is quite abundant in the brain, also have sleep-promoting effects (Imeri and Opp, 2009; Besedovsky et al., 2019). On the other hand, anti-inflammatory cytokines, such as IL-10, appear to attenuate NREM sleep. Furthermore, IFN has been associated with a decrease in REM sleep (Besedovsky et al., 2019). Curiously, many of these cytokines fluctuate during the day, with TNF and IL-6 for example peaking during sleep (Besedovsky et al., 2019).

Sleep disturbances are a characteristic symptom of the late stage of sleeping sickness (Brun et al., 2010). As mentioned above, unlike many infections which cause hypersomnia, sleeping sickness is not simply characterized by an increase in sleep, but instead affects both the timing of sleep and sleep architecture (Buguet et al., 2005).

### Timing of Sleep

Patients' sleep during daytime increases making it an obvious feature of the disease. However, these patients also experience insomnia at night (Buguet et al., 1999, 2001).

### Sleep Architecture

There is documentation of SOREM (sleep onset rapid eye movement) episodes in sleeping sickness patients that are similar to those seen in narcolepsy patients (Buguet et al., 2001). SOREM episodes are a sudden abnormal transition from wake to REM sleep, or after an extremely short NREM sleep period (Figure 3). *T. gambiense* infection shares other features with narcolepsy besides SOREM episodes, such as excessive daytime sleepiness and sleep fragmentation. It is possible that some of these symptoms are due to decreased levels of the wake-promoting orexin neuropeptide (see Bentivoglio and Kristensson, 2007; Dauvilliers et al., 2008).

*Trypanosoma brucei* parasites accumulate in regions of the brain involved in sleep regulation, and, in response to infection, there is massive infiltration of inflammatory cells and recruitment of activated astrocytes and microglial cells in these regions as well (Lundkvist et al., 2004). It is likely that this plays a role in the deregulation of sleep in sleeping sickness infection, particularly in the disruption of sleep architecture. Interestingly, despite the high inflammation in the brain (Sternberg et al., 2005), there is almost no neuronal degeneration reported in sleeping sickness patients (Kristensson et al., 2010). However, careful characterization in animal models of sleeping sickness showed there are decreased wake-promoting orexin neuron numbers, along with reduced dendrite branching and levels of orexin in the CSF (Palomba et al., 2015). Additionally, CSF orexin levels in sleeping sickness patients are lower than in healthy individuals, suggesting the involvement of this hypothalamic peptide in sleep architecture deregulation upon *T. brucei* infection, although levels are not as low as in narcoleptic patients (Dauvilliers et al., 2008). On a side note, it is curious that narcolepsy is now understood to be an autoimmune disorder. It has been even shown to be seasonal and to have had a spike in episode numbers following the 2009 H1N1 influenza pandemic (Han et al., 2011). These diseases may be indeed connected and we may learn more in the future about sleeping sickness also from the advances in the narcolepsy research. Nonetheless, loss of orexin neurons cannot explain all of the symptoms of sleeping sickness, since patients' sleep disruption appears to be reversed upon treatment (Mpandzou et al., 2011). Interestingly, prostaglandin D2 (PGD2), which induces NREM sleep, is significantly increased in the CSF of late stage sleeping sickness patients (Pentreath et al., 1990). Trypanosomes produce PGD2 for cell density regulation, which could interfere with homeostatic sleep regulation (Kubata et al., 2007); however, further studies are needed to assess if this contributes to the physiological deregulation observed in sleeping sickness patients, since these patients do not experience overall increases of NREM.

**Figure 4:**
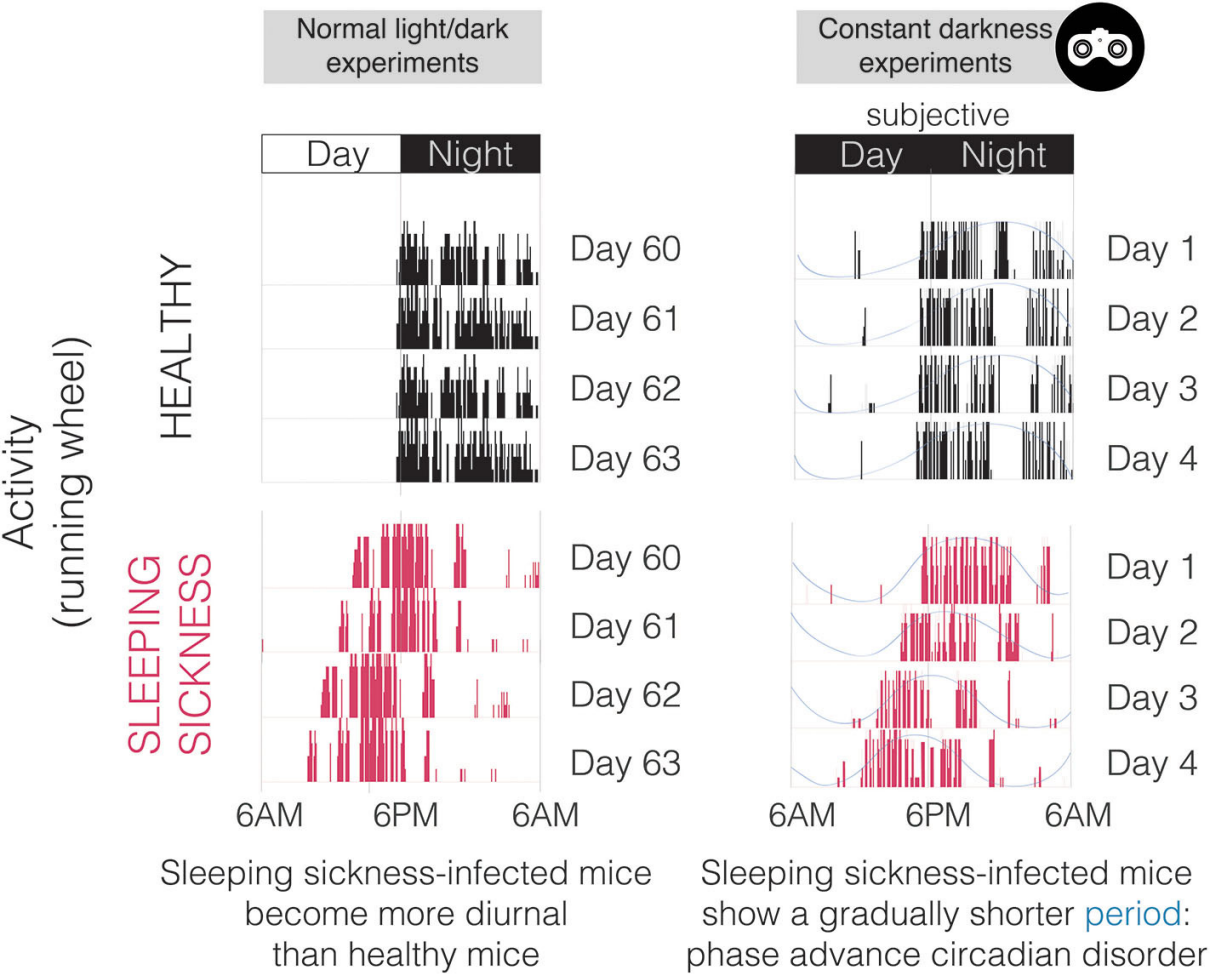
Activity plots (actograms) of both healthy and *T. brucei*-infected mice in normal light/dark conditions **(left)** and in constant darkness **(right)**. Note that this representative infected mouse does not run exclusively during the night period, which is extremely uncommon since mice are nocturnal and light imposes a very strong inhibitory effect on circadian behavior. In constant darkness (monitored with infrared goggles as represented on the top right), it is obvious that the period of activity is shorter in infected mice, especially when noting that the time at which activity starts (phase) becomes earlier every day.

**Figure 5:**
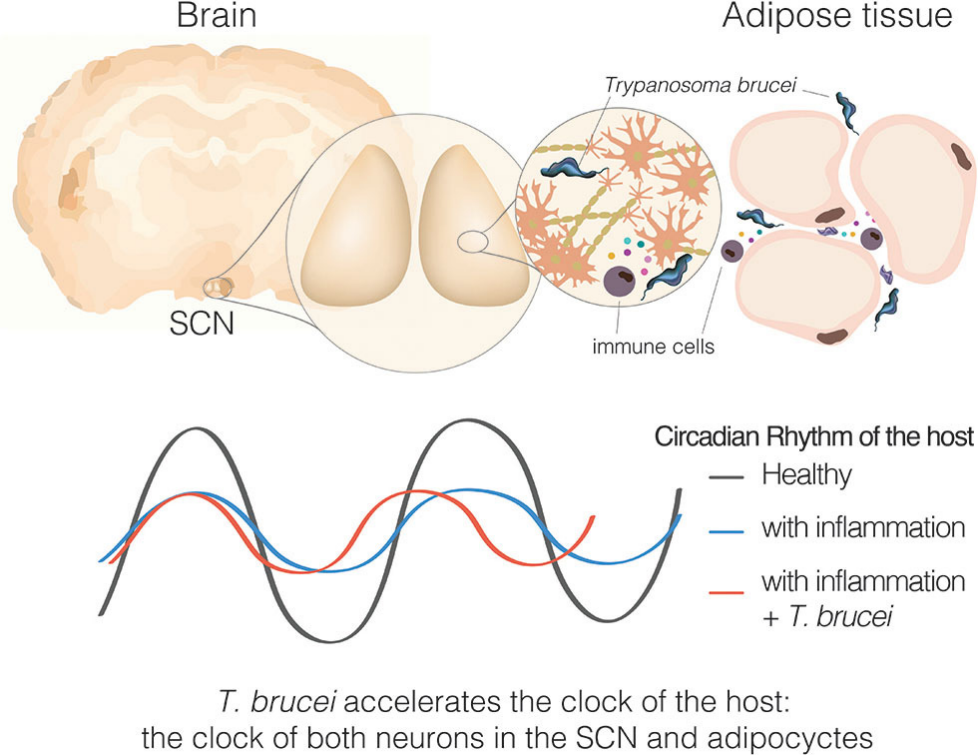
Sleeping sickness is a circadian disorder. Representation of a coronal brain section of a mouse, parasites, and inflammatory cells producing cytokines in response to the parasite presence. Bottom section represents the circadian rhythms of the host, either the behavioral output or molecular clock rhythms in SCN tissue or adipose tissue explant.

From our perspective, neuroinflammation can explain many of the symptoms of this disease, but perhaps not all of them. We recently demonstrated in a mouse model of sleeping sickness that *T. brucei* infection causes a specific circadian change: period shortening (Rijo-Ferreira et al., 2018) (Figures 4, 5). This acceleration of the clock originated molecularly, was observed in changes in rhythms from individual cells and tissues, and was manifested through altered behavior and core body temperature. The host molecular clock is composed of interconnected loops of transcriptional activators and repressors which are fine-tuned to make the clock tick with a period of 24 h (Takahashi, 2017; Rijo-Ferreira and Takahashi, 2019). Sleeping sickness infection accelerates this system; however, which circadian molecule(s) the infection alters remain unknown. The fact that circadian mouse behavior is altered by *T. brucei* infection indicated that the central clock in the SCN in the brain was affected. This is further supported by previous reports showing lower intrinsic SCN electrical activity from brain slices of infected rats (Lundkvist et al., 2002), and more recently with SCN explants from infected mice showing a shorter circadian period length of the molecular clock (Rijo-Ferreira et al., 2018). This detailed molecular characterization would be extremely difficult to study in humans; however, there have been indirect observations that suggest an acceleration of the clock in sleeping sickness patients. The pineal hormone melatonin, which anticipates the daily onset of darkness, remains cycling in sleeping sickness patients in the late stage, although it is secreted earlier than expected (i.e., is phase advanced, similar to what we observe in tissues in the mouse model, Figures 4, 5) (Claustrat et al., 1998). Since, so far, this period shortening appears to be unique to sleeping sickness and not a common feature of other infections or inflammatory stimuli, it could potentially be the result of molecule(s) produced by the parasite or by the host in response specifically to *T. brucei* parasitic infection.

## A Circadian Clock in *T. brucei*

One recent plot twist is that the protozoan parasite that disrupts the host clock has a circadian clock itself (Rijo-Ferreira et al., 2017a). Multiple organisms across the tree of life have been described to have circadian clocks, with key aspects of the molecular mechanism identified from bacteria, fungus, plants and animals (Saini et al., 2019). However, prior to our work there was no concrete evidence for the existence of circadian clocks in parasitic protozoans (Rijo-Ferreira et al., 2017b). Nonetheless, many observations of rhythms had been described: (i) transmissible forms of filarial parasites notoriously migrate from the lymphatic system and lungs into the bloodstream at specific times of the day (Thurston, 1951); (ii) malaria parasites burst red blood cells upon the completion of their cell-cycle leading to waves of fevers (Hawking et al., 1966); (iii) human infectious forms of the *Schistosoma* parasite (known as cercariae forms) emerge from snails during the daytime and swim in fresh water to infect the host by penetrating through their skin (Theron, 1989); (iv) Even trypanosomes (*T. rotatorium, T. congolense* and *T. lewisi*) have been described to fluctuate their numbers daily in the blood of their hosts (Southworth et al., 1968; Cornford et al., 1976; Hawking, 1978). However, what has remained unclear is whether these daily rhythms are driven by parasite biology or merely a consequence of rhythms within the host (Figure 2).

Since *T. brucei* is an extracellular parasite that can be easily cultured across different life cycle stages, it provided us with an excellent model to test whether parasites have an intrinsic clock. We found that both the bloodstream slender forms and procyclic forms have intrinsic rhythms of gene expression, most likely driven by a common mechanism (Rijo-Ferreira et al., 2017a; Figure 1). Thrillingly, how it does so is unlikely to be similar to previously described molecular clocks for other organisms. Although clocks are conserved features across evolution, they appear to have evolved multiple times, with the key clock components of each organism being different (convergent evolution, Figure 2). In addition, while it seems that transcriptional feedback is at the core of most clocks, this is unlikely to be the case in *T. brucei* since most of its gene expression regulation is at the posttranscriptional level (Box 1) (Siegel et al., 2011). Instead of transcriptional activators and repressors (as in the core of most clock mechanisms described so far), a posttranscriptional clock in *T. brucei* will likely rely on the regulation of mRNA decay, RNA binding proteins, and other posttranscriptional regulatory pathways. What those may be is still unknown and thus, the quest to discover clock genes in *T. brucei* will continue.

Box 1 **Curiosities of sleeping sickness infection and research.**

***Antigenic variation***

*T. brucei* evasion of the immune system is extremely sophisticated. Parasites are able to frequently switch their glycoprotein coat, continuously making the antibody response of the host obsolete and evading clearance (Mugnier et al., 2015). An incredible repertoire of >1,000 genes encodes these glycoprotein coats (known as VSG, variant surface glycoprotein), and there is further potential for VSG mosaics (mix and match forms of other VSG genes) to form (Mugnier et al., 2016). Malaria parasites also have a similar antigenic variation strategy to escape the host's immune system.

***Unorthodox genome organization***

Most *T. brucei* genes are encoded in enormously large polycistronic units with many genes under one single promoter that are constitutively transcribed (Siegel et al., 2011). Although commonly seen in bacteria, this is quite unusual for eukaryotes. Thus, unlike most eukaryotes, in *T. brucei* it is believed that gene expression is mostly regulated at the posttranscriptional level and that there is little transcriptional control (Siegel et al., 2011). In addition to its 11 pairs of chromosomes, the *T. brucei* genome also has an additional ~5 intermediate-size chromosomes and ~100 mini chromosomes (encoding many of the VSGs) (Daniels et al., 2010).

***DNA base J***

Base J is a Kinetoplastid-specific DNA hypermodification of thymine, T (hydroxylation and then glycosylation) that was initially described in *T. brucei* (Bernards et al., 1984; Pays et al., 1984). In *Leishmania* it appears to function as a termination signal of transcription (van Luenen et al., 2012).

***Stripes in zebras***

Why do zebras have black and white stripes? It is now believed to be (in part) the result of an evolutionary advantage to avoid the biting of tsetse flies. Studies have shown that tsetse flies and other obligate blood-feeding flies, are less likely to land on black and white striped surfaces than on uniform ones (Caro et al., 2014).

***Blood-brain barrier***

Attempts to deliver drugs to the brain for treatment of sleeping sickness, using an anti-trypanosome dye named trypan-blue, led to the discovery of the blood brain barrier (Bentivoglio and Kristensson, 2014). This dye has also become widely used to evaluate cell viability.

***RNA editing***

Post-transcriptional changes in the sequence of the RNA, known as RNA editing, exists across eukaryotes. In mammals, the most common RNA edit is adenosine-to-inosine. Curiously, the first documentation of RNA editing was in the cytosine c oxidase of *T. brucei* (Benne et al., 1986). This and the blood-brain barrier discovery are examples of how studying less prevalent, neglected parasitic infections can still uncover knowledge invaluable to the advancement of science.

Although the mechanisms of parasite rhythms need further investigation, it seems that they modulate (probably among other biology aspects) parasite metabolism. Interestingly, the susceptibility of the parasite to suramin, a drug used to treat the early stage of sleeping sickness, fluctuates 2.5-fold throughout the day *in vitro* (Rijo-Ferreira et al., 2017a), possibly due to the actions of suramin on parasite metabolic pathways (Alsford et al., 2012). This suggests that there may be a benefit to considering the timing of treatment of this disease (Rijo-Ferreira and Takahashi, 2019).

## What Could be the Evolutionary Advantage for Such Sleep and Circadian Deregulation?

It is a mystery why *T. brucei* causes a disease with symptoms such as sleep and circadian disruption. To reflect on it, one must consider all three parties involved: vector, parasite, and host. Curiously, there is evidence that *T. brucei* infection modulates tsetse feeding behavior, making it last longer by modulating the anti-hemostatic properties of the saliva. This is thought to increase the chances of transmission upon the blood-meal and is also associated with higher host-seeking behavior by the tsetse (Van Den Abbeele et al., 2010). It seems plausible to assume that sleepiness/napping during the daytime may also increase the chances of trypanosomes to be transmitted from the host to the insect. Tsetse flies are diurnal (Brady and Crump, 1978), they bite outdoors in the savannah, and are attracted to movement, so perhaps a debilitating hypersomnia type of symptom would prove rather ineffective to propagate the disease. However, these flies are also pool feeders, i.e., they cut the skin of mammals and suck up blood from the respective lesion (Van Den Abbeele et al., 2010). The bite is rather painful and sufficient feeding takes time. Humans can reach any part of the body with their hands, therefore feeding would be more effective if the person does not react temporarily, as in a short sudden nap during the daytime.

Perhaps most of the advantage comes from the circadian deregulation of the host: such as the attempt of interfering with the very sophisticated and well-orchestrated immune response to pathogenic invaders, or the host metabolism to “feed” the quick and demanding replication of the parasite? This may not be the case since these would be advantageous to many other human pathogens, and, so far, no others have been shown to lead to such a specific acceleration of the circadian clock.

Nonetheless, there are a couple other examples of modulation of the circadian behavior by non-mammalian pathogens. The fungus *Ophiocordyceps unilateralis s.l*. infects nighttime carpenter ant workers. When the fungus is grown enough it makes the ant wander out of the nest during daytime and seek elevated vegetation. The “zombie” ant dies, and the fungus completes its life cycle releasing spores from within the ant (de Bekker et al., 2015). Anecdotal observations suggest that there is also a time-of-day specificity for the “suicide” of crickets that are infected with hairworms (Nematomorpha). This jumping of the insect into an aquatic environment, which is needed for the completion of the worm's life cycle, seems to occur mostly during the early night (Thomas et al., 2002).

What is even more complex to understand is how to integrate both clocks of host and parasite. What happens to the parasite clock in the context of sleeping sickness, once host rhythms become disrupted? This will eventually abolish the regular circadian inputs the parasite population is presumably used to receiving. Does this impair the parasite population synchrony? Is this an advantage also to not kill the host? A long-standing idea in the parasitology field is that killing the host is never an ideal outcome for a parasite whose purpose is to ensure transmission. Also, if the parasite secretes molecules that modulate the rhythm of the host, is the fly's clock also affected? These questions and more remain to be answered.

## Final Remarks

The contribution of the research on sleeping sickness infection has been impactful also over multiple fields (Box 1). This makes the case that even if the disease becomes eradicated, the study of host-parasite-fly interactions is an important model to understand biology. It is notable that both sleep architecture and sleep/wake cycle disruption in sleeping sickness patients can be reversed upon treatment (Buguet et al., 1993; Figure 3). At the molecular level the same was observed, with treatment reversing the clock gene expression disruption in mice (Rijo-Ferreira et al., 2018). In addition, based upon autopsy studies, despite strong inflammatory response and microglia activation, there does not seem to be massive neuronal damage (Lundkvist et al., 2004). These observations suggest that the presence of the parasite itself might be responsible for the symptoms observed in sleeping sickness patients.

These open questions focus interest not only on the mechanism of this fatal disease but also how the parasite modulates the circadian clock of mammals since this system regulates almost all levels of body physiology and because of the interest in identifying molecules that can modulate this clock (Rijo-Ferreira and Takahashi, 2019).

Thus, perhaps further study will bring a better understanding of circadian manipulation of parasites, potentially identifying molecules that module the mammalian clock for circadian medicine and also how pathogens interact with hosts.

## Author Contributions

All authors listed have made a substantial, direct and intellectual contribution to the work, and approved it for publication.

## Conflict of Interest

The authors declare that the research was conducted in the absence of any commercial or financial relationships that could be construed as a potential conflict of interest.
